# Chest CT in COVID-19 at the ED: Validation of the COVID-19 Reporting and Data System (CO-RADS) and CT Severity Score: A Prospective, Multicenter, Observational Study

**DOI:** 10.1016/j.chest.2020.11.026

**Published:** 2020-11-30

**Authors:** Arthur W.E. Lieveld, Kaoutar Azijli, Bernd P. Teunissen, Rutger M. van Haaften, Ruud S. Kootte, Inge A.H. van den Berk, Sabine F.B. van der Horst, Carlijn de Gans, Peter M. van de Ven, Prabath W.B. Nanayakkara

**Affiliations:** a Section General & Acute Internal Medicine, Department of Internal Medicine, Amsterdam Public Health Research Institute, Amsterdam UMC, location VUmc, Amsterdam, The Netherlands; b Section Emergency Medicine, Emergency Department, Amsterdam Public Health Research Institute, Amsterdam UMC, location VUmc, Amsterdam, The Netherlands; c Section Radiology, Department of Radiology and Nuclear Medicine, Amsterdam UMC, location VUmc, Amsterdam, The Netherlands; d Section Acute Medicine, Department of Internal Medicine, Amsterdam UMC, location VUmc, Amsterdam, The Netherlands; e Section Radiology, Department of Radiology and Nuclear Medicine, Amsterdam UMC, location VUmc, Amsterdam, The Netherlands; f Department of Epidemiology & Data Science, Amsterdam UMC, location VUmc, Amsterdam, The Netherlands

**Keywords:** COVID-19, CT, emergency medicine, pneumonia, AUC, area under the curve, CO-RADS, COVID-19 Reporting and Data System, COVID-19, coronavirus disease 2019, CTSS, CT severity score, CUSUM, cumulative sum control chart, MDT, multidisciplinary team of experts, NVvR, Radiological Society of the Netherlands, PCR, polymerase chain reaction, WHO, World Health Organization

## Abstract

**Background:**

CT is thought to play a key role in coronavirus disease 2019 (COVID-19) diagnostic workup. The possibility of comparing data across different settings depends on the systematic and reproducible manner in which the scans are analyzed and reported. The COVID-19 Reporting and Data System (CO-RADS) and the corresponding CT severity score (CTSS) introduced by the Radiological Society of the Netherlands (NVvR) attempt to do so. However, this system has not been externally validated.

**Research Question:**

We aimed to prospectively validate the CO-RADS as a COVID-19 diagnostic tool at the ED and to evaluate whether the CTSS is associated with prognosis.

**Study Design and Methods:**

We conducted a prospective, observational study in two tertiary centers in The Netherlands, between March 19 and May 28, 2020. We consecutively included 741 adult patients at the ED with suspected COVID-19, who received a chest CT and severe acute respiratory syndrome coronavirus 2 (SARS-CoV-2) PCR (PCR). Diagnostic accuracy measures were calculated for CO-RADS, using PCR as reference. Logistic regression was performed for CTSS in relation to hospital admission, ICU admission, and 30-day mortality.

**Results:**

Seven hundred forty-one patients were included. We found an area under the curve (AUC) of 0.91 (CI, 0.89-0.94) for CO-RADS using PCR as reference. The optimal CO-RADS cutoff was 4, with a sensitivity of 89.4% (CI, 84.7-93.0) and specificity of 87.2% (CI, 83.9-89.9). We found a significant association between CTSS and hospital admission, ICU admission, and 30-day mortality; adjusted ORs per point increase in CTSS were 1.19 (CI, 1.09-1.28), 1.23 (1.15-1.32), 1.14 (1.07-1.22), respectively. Intraclass correlation coefficients for CO-RADS and CTSS were 0.94 (0.91-0.96) and 0.82 (0.70-0.90).

**Interpretation:**

Our findings support the use of CO-RADS and CTSS in triage, diagnosis, and management decisions for patients presenting with possible COVID-19 at the ED.

**Take-home Points**

**Research Question:** We aimed to prospectively validate the coronavirus disease 2019 (COVID-19) Reporting and Data System (CO-RADS) at the ED to be able to compare COVID-19 CT data across different settings and countries. We also evaluated whether the corresponding CT severity score (CTSS) was associated with prognosis.

**Results:** We observed that CO-RADS had an area under the curve compared with severe acute respiratory syndrome coronavirus 2 (SARS-CoV-2) polymerase chain reaction (PCR) of 0.91 (CI, 0.89-0.94). After correcting for confounders, the CTSS was significantly positively associated with hospital and ICU admission and mortality. We observed good-to-excellent interobserver agreement for CO-RADS and CTSS and no steep learning curve.

**Interpretation:** Our findings support the use of CO-RADS and CTSS in triage, diagnosis, and management decisions for patients presenting with suspected COVID-19 at the ED.

FOR EDITORIAL COMMENT, SEE PAGE 906

The coronavirus disease 2019 (COVID-19) pandemic continues to put tremendous stress on health-care systems and societies worldwide. With second waves flaring up globally,^1^^,^^2^ swift and accurate diagnosis is essential to profile patients and allocate scarce resources adequately. This is still hampered by limited sensitivity and availability of severe acute repiratory syndrome coronavirus 2 (SARS-CoV-2) polymerase chain reaction (PCR).^3^, ^4^, ^5^ More importantly, PCR does not give any insight into pulmonary involvement, whereas pneumonia is the most common cause of severe morbidity and mortality in COVID-19.^6^^,^^7^

Chest imaging may play a key role in COVID-19 triage and diagnosis, as well as stratification of disease severity.^8^, ^9^, ^10^ Conventional chest radiography unfortunately has limited sensitivity for COVID-19 pneumonia. In retrospective studies, sensitivity of chest CT for COVID-19 is excellent, and it may even be greater than that of PCR.^10^, ^11^, ^12^ CTs are helpful in the diagnostic process at the ED, because the results are available almost immediately, and alternative diagnoses may be identified. In addition, using a semiquantitative CT severity score (CTSS) is reported to correlate with disease severity, and it might be used as a prognostic marker.^12^, ^13^, ^14^, ^15^, ^16^, ^17^

Studies on using CT as an initial diagnostic modality are mostly retrospective in nature and from China, possibly reducing their generalizability to other regions. Although the CT characteristics of COVID-19 have been documented properly, no universal agreement exists on a systematic and reproducible way of evaluating and reporting CT abnormalities in patients with (suspected) COVID-19, which makes it difficult to compare data across different settings.^8^^,^^9^^,^^18^, ^19^, ^20^, ^21^

The Radiological Society of the Netherlands (NVvR) recently introduced such a method, the COVID-19 Reporting and Data System (CO-RADS), which is largely based on the recommendations of the Radiological Society of North America.^9^^,^^20^^,^^22^ The CO-RADS is meant to be used for patients with moderate to severe symptoms of (suspected) COVID-19. It employs a scoring system from 0 to 5 to classify pulmonary involvement from very unlikely to very likely, respectively (e-Table 1). In 105 patients, the NVvR found a very good performance for predicting COVID-19 (area under the curve [AUC] of 0.91 (CI, 0.85-0.97).^20^ In addition, interobserver agreement was substantial. Although these results are encouraging, a prospective external validation of the CO-RADS or any other CT classification system for COVID-19 is lacking.

**Table 1:** Patient Characteristics

Patient Characteristics	All Patients (N = 741)	SARS-CoV-2 PCR Positive (n = 235)	SARS-CoV-2 PCR Negative (n = 506)	*P* Value
Age, mean (SD)	62.1(17.2)	62.5 (14.6)	61.9 (18.4)	.655
Male, No. (%)	417 (56.3)	136 (57.9)	281 (55.5)	.209
Admission, No. (%)	580 (78.3)	208 (88.5)	372 (73.5)	**.000**
Admission IC, No. (%)	84 (11.3)	44 (18.7)	40 (7.9)	**.000**
30 days mortality, No. (%)	74 (10.0)	33 (14.0)	41 (8.1)	**.001**
In-hospital mortality, No. (%)	54 (7.3)	28 (11.9)	26 (5.1)	**.000**
Duration of symptoms, days (SD)	6.7 (7.2)	7.9 (4.6)	6.0 (8.2)	**.001**
Co-morbidities, No. (%)				
Asthma	49 (6.6)	16 (6.8)	33 (6.5)	.77
Chronic cardiovascular disease	197 (26.6)	50 (21.3)	147 (29.1)	**.000**
COPD (GOLD > 2)	128 (17.3)	27 (11.5)	101 (20.0)	**.000**
Current malignancy	107 (14.4)	15 (6.4)	92 (18.2)	**.000**
Diabetes mellitus	189 (25.5)	69 (29.4)	120 (23.7)	**.002**
Hypertension	292 (39.4)	107 (45.5)	185 (36.6)	**.000**
Observations and laboratory results at presentation				
CRP (mg/L), median (IQR)	44.0 (86.5)	69.0 (86.0)	28.0 (79.3)	.76
PCT	0.1 (0.28)	0.13 (0.22)	0.1 (0.35)	**.002**
Positive blood culture, No. (%)	36 (4.9)	1 (0.4)	35 (6.9)	**.000**
Modified early warning score, mean (SD)	2.86 (1.8)	3.0 (1.7)	2.8 (1.8)	.076
Temperature (°C), mean (SD)	36.9 (1.3)	37.5 (1.1)	36.7 (1.3)	.259
Respiratory rate, mean (SD)	22.7 (7.9)	24.9 (8.0)	21.7 (7.7)	.070
Saturation levels, mean (SD)	95.2 (5.6)	94.1 (6.1)	95.7 (5.3)	.281
Oxygen therapy, No. (%)	255 (34.9)	111 (47.8)	144 (28.9)	**.002**
Intubation, No. (%)	68 (9.2)	39 (16.6)	29 (5.7)	**.000**

*P* values in bold are < .05.

CRP = C-reactive protein; GOLD = Global Initiative for Chronic Obstructive Lung Disease; IC = intensive care; IQR = interquartile range; PCR = polymerase chain reaction; PCT = procalcitonin; SARS-CoV-2 = severe acute respiratory syndrome coronavirus 2.

In this observational study, we therefore set out to prospectively validate the CO-RADS in two tertiary hospitals in The Netherlands. Furthermore, in accordance with recent research recommendations from the World Health Organization (WHO),^23^ we analyzed whether the CTSS was associated with hospital admission, ICU admission, and mortality.

## Methods

### Study Protocol and Design

This is a real-life, prospective, observational study. Patients were recruited consecutively from the EDs of the two university hospitals in Amsterdam, the Netherlands, between March 19 and May 28, 2020. The study was approved by the Medical Ethics Review Committee of the Amsterdam University Medical Centers, location VUmc. An opt-out recruitment procedure was used to obtain consent for participation in the study.

**Figure 1:**
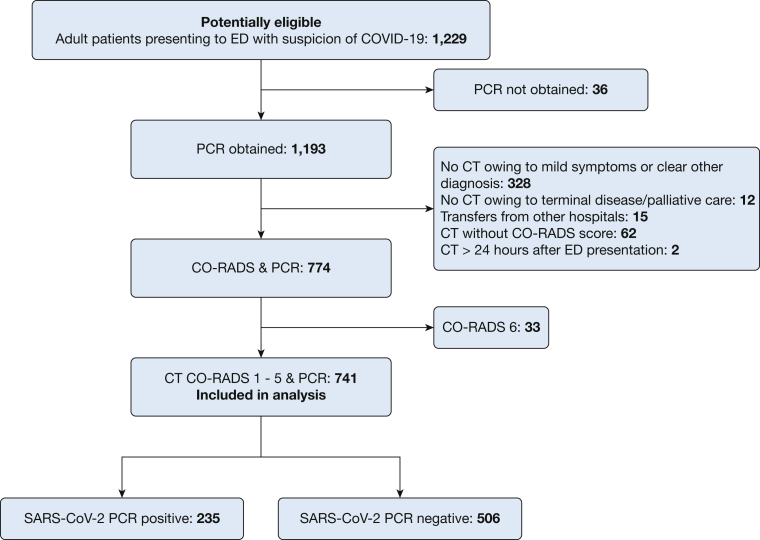
Study population diagram. CO-RADS = COVID-19 Reporting and Data System; PCR = polymerase chain reaction.

All patients 18 years of age and older who visited the ED with suspected COVID-19 according to the WHO and Dutch Centre for Infectious Disease Control case definition (including either fever, malaise, respiratory symptoms, GI symptoms, or loss of taste and smell) were potentially eligible. Patients were included if they received a PCR and chest CT with CO-RADS score. Exclusion criteria were age younger than 18 years, unwillingness to give verbal consent, or a CO-RADS 6 score (Fig 1).

### Medical Workup

All patients with suspected COVID-19 received a regular medical workup and a chest CT if deemed appropriate by the treating physician or according to local clinical protocol. Local protocol, based on Dutch Federation of Medical Specialists and WHO COVID-19 recommendations, dictated that physicians could perform a CT in a case of moderate to severe symptoms.^23^^,^^24^ In a case with mild symptoms, physicians could forego additional imaging. Hospital admission criteria were saturation < 94% or respiratory rate > 20 breaths/min. ICU admission criteria were deterioration despite conventional respiratory support, patients requiring mechanical ventilation, or multi-organ failure. Admission decisions were not made based on CT data. All admitted patients with (suspected) COVID-19 were discussed daily in a multidisciplinary team (MDT), consisting of consultants in infectious disease, respiratory disease, and microbiology. The MDT decided on the final diagnosis, but it was not blinded for the CT results.

### CT

The CTs were assessed by our local radiologists with varying degrees of experience. The radiologists had regular access to clinical information but did not have access to the PCR results, because CT scanning and reporting were done early after ED admission, before PCR results were available. The likelihood of COVID-19 pneumonia was reported according to the CO-RADS classification system (e-Table 1).^20^^,^^22^ Details on how the CTs were made are available in the e-Appendix under e-Methods.

To quantify pulmonary involvement, every CT with a CO-RADS of 3 or higher was graded according to the CTSS.^25^ This is a visual assessment of the percentage of disease involvement in each lobe (e-Table 2). The total CTSS is the sum of the individual lobar scores and can range from 0 (no involvement) to 25 (maximum involvement).

### PCR

A naso- or oropharynx PCR was performed in all patients presenting to the ED, according to WHO standards. In case of a negative or inconclusive test result and high clinical suspicion of COVID-19, a nasopharynx or oropharynx PCR was repeated or sputum or bronchoalvealar lavage if available and possible, as indicated by the MDT.

### Outcomes

As the primary outcome, sensitivity, specificity, predictive values, likelihood ratios, area under the receiver operating characteristic curve, and diagnostic accuracy were calculated for CO-RADS. For CTSS, we calculated the same measures, and ORs for hospital admission, ICU admission, and mortality.

### Statistical Analysis

Continuous variables were summarized by mean and SD, or by medians and interquartile range. Differences between groups were tested using the independent-samples *t* test, Mann Whitney *U* test, χ2 test or Fisher exact test as appropriate. A two-sided significance level of 5% was used, and 95% CIs were reported for all analyses.

Discriminatory ability of CO-RADS and CTSS was determined by the area under the receiver operating characteristic curve. The optimal cutoff value was determined by the Youden’s index. Diagnostic measures were subsequently calculated.For CTSS, we also employed a “gray zone” approach. A gray zone represents a predictive test of poor accuracy, with a sensitivity and specificity <90%. Sensitivity and specificity were plotted as a function of the CTSS, to determine a gray zone for hospital admission, ICU admission, and mortality.

Logistic regression analysis was performed to test whether the CTSS score was associated with hospital admission, ICU admission, and 30-day mortality. Associations were quantified by ORs. We corrected for the following potential confounders: age, sex, and co-morbidities (COPD, asthma, cardiovascular disease, hypertension).^12^, ^13^, ^14^, ^15^, ^16^, ^17^

Interobserver agreement was quantified between two acute radiologists, with 3 and 6 years of experience, and the initial assessing radiologist via the intra-class correlation coefficient. They independently assessed the CT images of 63 patients.

Cumulative sum control (CUSUM) charts were used to assess the learning curve for using CO-RADS for diagnosis of COVID-19. Two separate CUSUM charts were made—one for patients with a negative PCR (non-COVID) and one for patients with a positive PCR (COVID). In addition, CUSUM charts were made for overall accuracy including all patients.

## Results

From March 19 until May 28, 2020, 1,229 patients with symptoms suggesting COVID-19 presented at the two EDs. Seven hundred forty-one patients fulfilled the inclusion criteria, of which 235 had a positive PCR result (see e-Table 3 for details on the excluded patients). In 56 of the PCR-positive patients, the initial PCR was negative, and repeated PCR testing was needed to confirm COVID-19. Patients in the PCR-positive group were on average more likely to require oxygen therapy, hospital admission, or ICU admission, and had a higher mortality rate. PCR-positive patients were also more likely to have comorbidities such as cardiovascular disease, hypertension, and COPD (Table 1).

### CO-RADS

**Figure 2:**
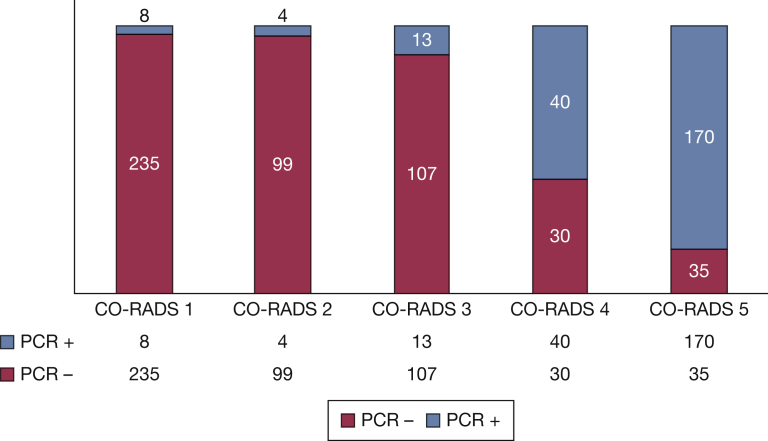
Distribution of PCR results and CO-RADS. CO-RADS = COVID-19 Reporting and Data System; PCR = polymerase chain reaction.

**Figure 3:**
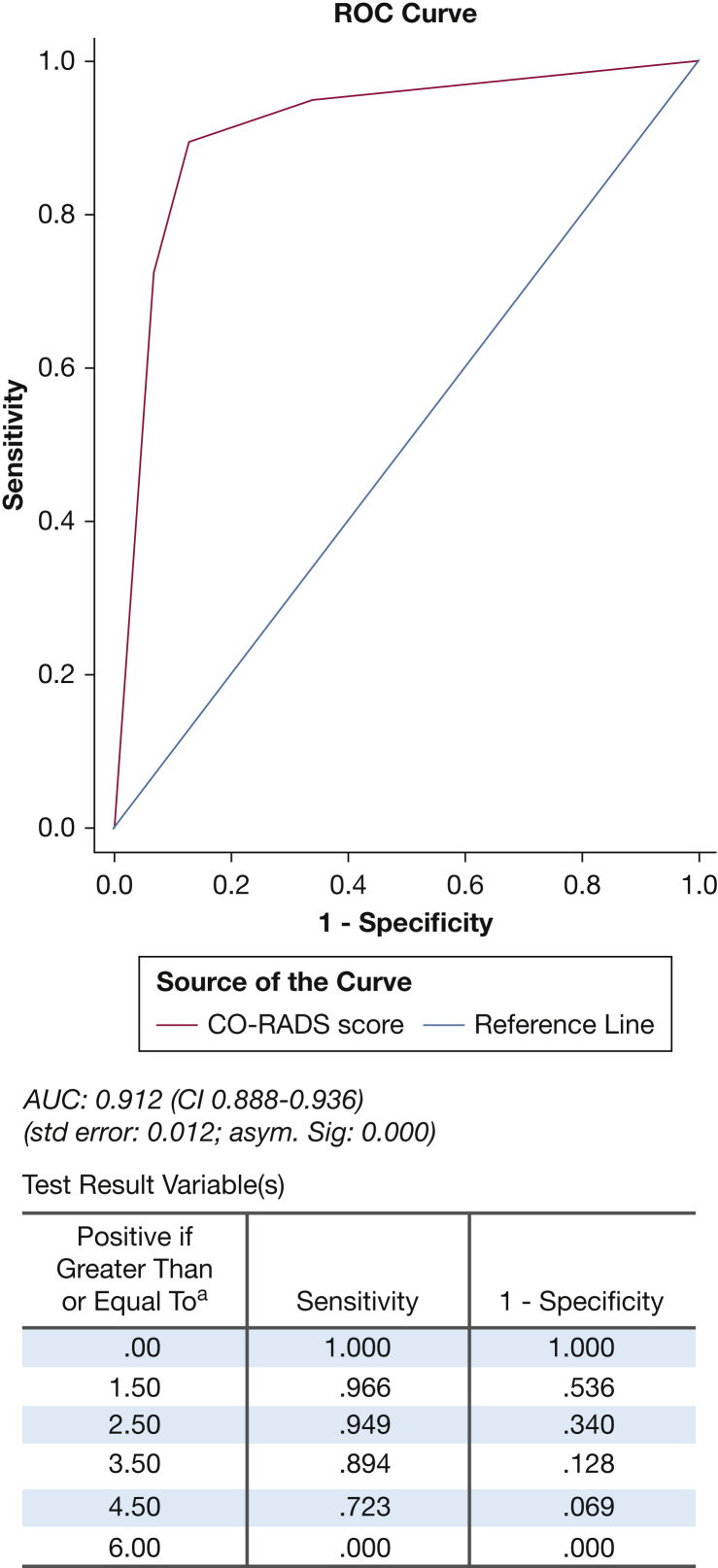
ROC Curve CO-RADS vs PCR results. AUC = area under the curve; CO-RADS = COVID-19 Reporting and Data System; PCR = polymerase chain reaction; ROC = receiver operating characteristic.

Figure 2 shows the distribution of PCR results with the CO-RADS score. We found that CO-RADS ≥ 4 was the optimal cutoff for discriminating between a positive and negative PCR, with an AUC of 0.91 (CI, 0.89-0.94), with a sensitivity of 89.4% (CI, 84.7-93.0), specificity of 87.2% (CI, 83.9-89.9), negative predictive value of 94.6% (CI, 92.4-96.2), and positive predictive value of 76.4% (CI, 71.9-80.3) (Fig 3; Table 2). The intraclass correlation coefficient was 0.94 (CI, 0.91-0.96).

**Table 2:** Diagnostic Accuracy Measures for CO-RADS

Diagnostic Accuracy Measures	CO-RADS 345 vs SARS-CoV-2 PCR	CO-RADS 45 vs SARS-CoV-2 PCR
Sensitivity, % (95%CI)	95.1 (91.6-97.4)	89.4 (84.7-93.0)
Specificity, % (95%CI)	66.0 (61.7-70.1)	87.2 (83.9-89.9)
Positive likelihood ratio	2.8 (2.5-3.2)	6.96 (5.2-8.8)
Negative likelihood ratio	0.07 (0.04-0.13)	0.12 (0.08-0.18)
Positive predictive value, % (95%CI)	57.5 (54.5-60.6)	76.4 (71.9-80.3)
Negative predictive value, % (95%CI)	96.5 (94.1-98.0)	94.6 (92.4-96.2)
Accuracy, %	75.5 (72.3-78.5)	87.9 (82.3-90.1)

CO-RADS = COVID-19 Reporting and Data System; SARS-CoV-2 = severe acute respiratory syndrome coronavirus 2.

Twenty-five patients had a false-negative CT scan (CO-RADS ≤ 3, but positive PCR). Patients with a CO-RADS ≤ 2 had a significantly shorter duration of symptoms than those with a CO-RADS >2 (e-Table 4). Eleven (44%) of them had a symptom duration of less than 3 days. Eighteen patients (72%) were admitted, seven (28%) patients received oxygen, and only two (8%) patients needed ICU treatment. Sixty-five patients had a false-positive CT scan (CO-RADS ≥ 4, but negative PCR), compared with PCR. Forty-six of those patients were still classified as having COVID-19 by the MDT; the remaining diagnoses are summarized in e-Table 5. The most common alternative diagnoses were bacterial pneumonia and decompensated heart failure (e-Table 6).

The CUSUM plots in center 1 showed no strong upward or downward inflections, indicating a steady performance over time. For center 2, an upward inflection was seen after the 80^th^ COVID-negative patient was included (corresponding to the 161^th^ consecutive patient included), showing a temporary increase in the false-positive rate (e-Fig 5).

### CTSS

The CTSS was determined in 304 (patients, who had a CO-RADS ≥ 3. Stratified according to three disease severity groups—(1) discharge home from ED, (2) hospital admission, or (3) ICU admission—we found a significant difference in mean CTSS; 5.5 (SD, 3.6), 9.4 (SD, 4.9), and 14.8 (SD, 4.8), respectively.

Logistic regression showed a significant positive association between CTSS (per point increase) and hospital admission, ICU admission, and 30-day mortality (Table 3). These associations stayed significant after correcting for potential confounders age, sex, and the aforementioned co-morbidities, with adjusted OR of 1.18 (CI, 1.09-1.28); 1.23 (CI, 1.15-1.32); and 1.14 (CI, 1.07-1.22) respectively.

**Table 3:** CTSS Logistic Regression

Association between CT-severity score and hospital admission				
	Crude Analysis		Adjusted Analysis^a^	
	OR (95% CI)	*P* Value	OR (95% CI)	*P* Value
CT severity score	1.18 (1.09-1.27)	.000	1.18 (1.09-1.28)	.000

Association between CT-severity score and ICU admission				
	Crude Analysis		Adjusted Analysis^a^	
	OR (95% CI)	*P* Value	OR (95% CI)	*P* Value
CT-severity score	1.23 (1.16-1.32)	.000	1.23 (1.15-1.32)	.000

Association between CT-severity score and 30-day mortality				
	Crude Analysis		Adjusted Analysis^a^	
	OR (95% CI)	*P* Value	OR (95% CI)	*P* Value
CT-severity score	1.12 (1.05-1.19)	.001	1.14 (1.07-1.22)	.000

OR and CI of the association between CT-severity score (CTSS) and hospital admission, ICU admission, and 30-day mortality per point increase in CTSS.

a Adjusted for age, sex, co-morbidities (cardiovascular disease, COPD, asthma, high BP).

For hospital admission, the optimal cutoff of the CTSS was 9, with an adequate AUC of 0.77 (CI, 0.71-0.84), a sensitivity of 65.4% (CI, 59.3-71.1), and specificity of 78.1% (CI, 62.4-89.4). For ICU admission CTSS had a good AUC of 0.81 (CI, 0.75-0.88) at a cutoff of 13, with a sensitivity of 72.6% (CI, 58.3-84.1), and specificity of 79.8% (CI, 74.4-84.6). For 30-day mortality, CTSS had a poor AUC of 0.63 (CI, 0.53-0.73) at a cutoff of 13, with a sensitivity of 72.2% (CI, 58.3-84.1) and specificity of 79.8% (CI, 74.4-84.6) (e-Figs 1-3).

Gray zone analysis showed that a CTSS ≥10 was predictive of hospital admission with a specificity of ≥90%, whereas a score of ≤3 was ≥90% sensitive for ED discharge. A CTSS ≥15 was predictive of ICU admission with a specificity of ≥90%, and a score ≤9 excluded ICU admission with a sensitivity of ≥90%. A score of ≥17 was ≥90% specific for 30-day mortality, and a score ≤5 excluded 30-day mortality with a sensitivity of ≥90% (e-Fig 4a-c). The intra-class correlation coefficient for the CTSS was 0.82 (CI, 0.7-0.9).

## Discussion

To our knowledge, this is the first prospective multicenter study evaluating the diagnostic accuracy of chest CT in COVID-19 patients and externally validating a systematic CT reporting scheme. We found that CO-RADS discriminates excellently between a positive and negative PCR result, with an AUC of 0.91 (CI, 0.89-0.94). We also found that CTSS at ED presentation is associated with hospital admission, ICU admission, and 30-day mortality. The CUSUM analysis showed no significant learning curve. In combination with a high interobserver agreement, this indicates that the system is easy to learn and use, even for less experienced radiologists.

### CO-RADS

The high negative predictive values and low negative likelihood ratios associated with a CO-RADS ≤3 indicate that these scores can aid in excluding the presence of COVID-19. A score of ≤3 could also aid in patient management, particularly patient isolation measures. Interestingly, we found that almost half of the false-negative CTs were performed in patients with a short duration of symptoms (<3 days). Previous studies found that chest imaging might be negative in the earlier phases of the disease, because it has not involved the lung parenchyma yet.^11^ Scanning too early in the disease course could falsely reassure clinicians.

Our results indicate that a CO-RADS score ≥4 has a high positive likelihood ratio and a corresponding good positive predictive value in a high-prevalence setting. As was argued before by Ai et al^11^ and Prokop et al,^20^ the suboptimal clinical sensitivity of the gold standard PCR could result in a reduced specifity (and lower positive predictive value and positive likelihood ratio) because this generates more false-positive CTs.^3^^,^^5^ This would mean that specificity of CO-RADS might actually be higher. In any case, a CO-RADS ≥ 4 seems to ascertain the diagnosis of COVID-19. A score of ≥4 can therefore be used to put a patient in isolation or self-quarantine.

When COVID-19 prevalence decreases, however, or the prevalence of similar diseases increases, the positive predictive value of a high CO-RADS will likely decrease. A recent Chinese/American study suggests that CT can distinguish COVID-19 from other viral pneumonias with moderate to high accuracy.^26^ Further research is needed to assess whether CO-RADS is useful in a setting with a lower COVID-19 prevalence and a different case mix of other (viral) diseases that might produce similar abonormalities on CT.

### CO-RADS Summary

Our results externally validate CO-RADS as a reliable and reproducible tool in reporting pulmonary findings in patients with suspected COVID-19 in a pandemic setting. As stated in the Radiological Society of North America Consensus Statement,9 standardized reporting can provide guidance to radiologists, accelerate reporting during high demand, reduce reporting variability, enable care pathways, and facilitate a basis for data sharing, quality improvement, and research. CO-RADS can help achieve all those aims. Using CO-RADS could be especially helpful when PCR is not available and CT is conducted at the ED on patient arrival, as was the case in many Dutch hospitals during the pandemic. The Dutch Federation of Medical Specialists has incorporated CO-RADS in the national COVID-19 care pathway recommendation.^24^

### CTSS

Our results indicate that CTSS is positively associated with hospital and ICU admission, and also—albeit to a lesser extent—30-day mortality even after correcting for confounders. The positive association of CTSS and hospital or ICU admission is greater than that of mortality. We also observed a smaller gray zone for hospital/ICU admission than 30-day mortality. This difference is probably explained because the time-to-event in hospital/ICU admission is shorter than in 30-day mortality. Moreover, there are more complications of COVID-19 (ie, pulmonary embolism) that also affect mortality and thus are not captured by the CTSS.

However, although there are gray zones in each of these categories in which uncertainty remains, the upper and lower values can provide clinicians with a helpful tool to make faster and more adequate triage and management decisions, and to predict patient disease course. Especially because previous studies have shown that pulmonary involvement can predate worsening of severe respiratory symptoms,^13^^,^^14^ the CTSS could be particularly helpful in patients that do not seem clinically ill (eg, silent hypoxia), but based on the CTSS might deteriorate and thus benefit from admission and a higher level of monitoring.

### CT Use—Global Perspective

Although these results are encouraging, debate continues about the place of CT in COVID-19 diagnostics. Dutch and Chinese guidelines include CT in their workup of COVID-19, but American and British guidelines are more reserved.^8^, ^24^, ^27^ The recent Fleishner consensus statement concedes that the logistics surrounding the provision of CT services can be a daunting task in the face of this pandemic.^8^ As second waves are flaring up, this is of particular importance. CT is difficult to perform in unstable patients, and availability and costs can be an issue even in high-income countries. Risk of transmission during transport neccesitates stringent disinfection protocols and can lead to prolonged CT down time. We have seen significant health disparities in COVID-19 outcome along geographic, socioeconomic, ethnic, cultural, and racial lines.^28^, ^29^, ^30^, ^31^ Portable imaging modalities such as bedside lung ultrasound circumvent these issues, while showing better accuracy than CXR and similar accuracy to CT.^10^^,^^32^, ^33^, ^34^, ^35^, ^36^, ^37^, ^38^, ^39^, ^40^, ^41^, ^42^, ^43^ This may be considered as a viable alternative, especially in low-resource settings where CT might not be readily available.

### Limitations/Bias

Our study has some limitations. First, we stress that this study was conducted in a high-prevalence setting. Although CO-RADS was designed to be used in patients with moderate to severe symptoms in this pandemic, it is unclear how CO-RADS will function when the pandemic subsides, but it is reasonable to assume that the false-positive rate will increase in such a situation. Conversely, negative predictive value is likely to increase when disease prevalence decreases. However, we argue that the need for quick and reliable triage is more pressing in a pandemic setting than in a situation in which prevalence is low and medical resources are not as overwhelmed.

Second, we assessed diagnostic accuracy of CO-RADS and CTSS in a population presenting to ED that were deemed to have moderate to severe symptoms, which justified additional imaging of the chest. Although this might reduce generalizibility to all patients in all settings, the diagnostic accuracy measures we report are reflective of daily practice in EDs all over the globe. After all, physicians will be more likely to perform additional tests and exclude alternative diagnoses in patients with more severe symptoms than when symptoms are milder and less threatening.

Whether CO-RADS would be applicable in asymptomatic or mildly symptomatic patients outside the ED is unclear, but the question is whether this would be desirable. We would argue against the use of CT in the general outpatient population, because the possible benefits (early diagnosis, fast triage), probably do not outweigh the possible harms (overuse of medical resources, health-care costs, radiation, increase of false-negatives because of less pulmonary involvement in this group). Third, we believe selection bias was minimal; the treating physicians were not aware of this study being undertaken, so they were not influenced in whether their patient got a CT or not. The chance of nonrandom selection of patients is therefore low. In addition, we consecutively included a very large consecutive sample of COVID-19 patients. Fourth, information bias was minimized because the PCR results were not known at the time the CT was done, and if they were, the results were excluded from the analysis. Fifth, the CO-RADS and CTSS were assessed by radiologists with varying levels of experience. However, CUSUM analysis did not show clear learning effects in the participating hospitals. Combined with a high agreement, this would suggest that both CO-RADS and CTSS are easy to use and reproduce.

### Interpretation

CO-RADS is an easy-to-use tool to help radiologists and guide clinicians in diagnosis of COVID-19 in patients with moderate to severe symptoms presenting to the ED. Furthermore, the CTSS seems to correlate with hospital admission, ICU admission, and 30-day mortality, and therefore it might be used in management decisions. We encourage the international use of the CO-RADS and CTSS for patients with suspicion of COVID-19 at ED presentation, to provide a basis for further reporting, communication, and data sharing. We also suggest further studies to be conducted in other countries and care settings to increase the robustness of our findings.

## Supplementary Data

e-Online Data
