# Supplementary material for: Chest CT in COVID-19 at the ED: Validation of the COVID-19 Reporting and Data System (CO-RADS) and CT Severity Score: A Prospective, Multicenter, Observational Study
Source: Chest. 2020 Nov 30;159(3):1126–35. doi: 10.1016/j.chest.2020.11.026 (PMC7704067; doi:10.1016/j.chest.2020.11.026)
Supplement: e-Online Data [file mmc1.pdf]

CHEST Online Supplement

Chest CT in COVID-19 at the ED: Validation of the COVID-19 Reporting and Data System (CO-RADS) and CT Severity Score

A Prospective, Multicenter, Observational Study

Arthur W.E. Lieveld, MD; Kaoutar Azijli, MD; Bernd P. Teunissen, MD; Rutger M. van Haaften, MD; Ruud S. Kootte, MD, PhD; Inge A.H. van den Berk, MD; Sabine F.B. van der Horst, BSc; Carlijn de Gans, MD; Peter M. van de Ven, PhD; and Prabath W.B. Nanayakkara, MD, PhD

CHEST 2021; 159(3):1126-1135

Online supplements are not copyedited prior to posting and the author(s) take full responsibility for the accuracy of all data.

© 2020 AMERICAN COLLEGE OF CHEST PHYSICIANS. Reproduction of this article is prohibited without written permission from the American College of Chest Physicians. See online for more details. **DOI:** 10.1016/j.chest.2020.11.026

e-Figure 1: ROC curve CTSS vs Hospital admission

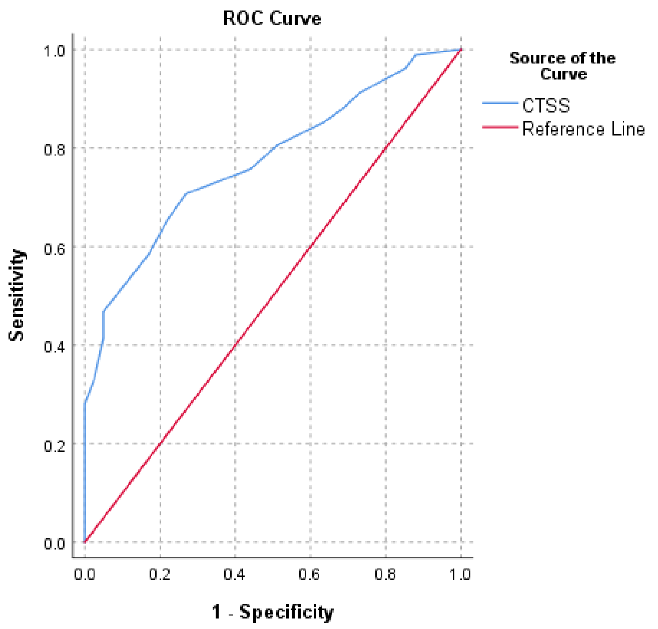

AUC: 0.773 (CI 0.710-836); ideal cut-off point: 9 (sens: 65.8; spec: 78.0)

e-Figure 2: ROC Curve CTSS vs ICU admission

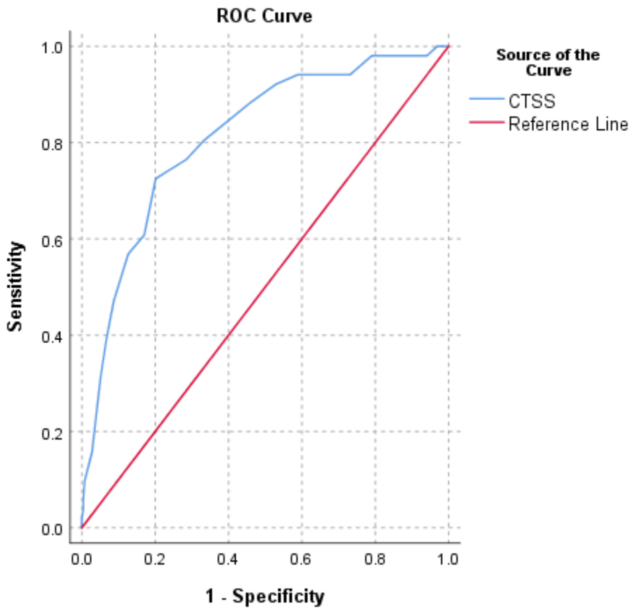

AUC: 0.81 (CI 0.745-0.876); Ideal cut-off point: 13 (sens: 72.2; spec: 79.6)

Online supplements are not copyedited prior to posting and the author(s) take full responsibility for the accuracy of all data.

e-Figure 3: ROC Curve CTSS vs 30-Day mortality

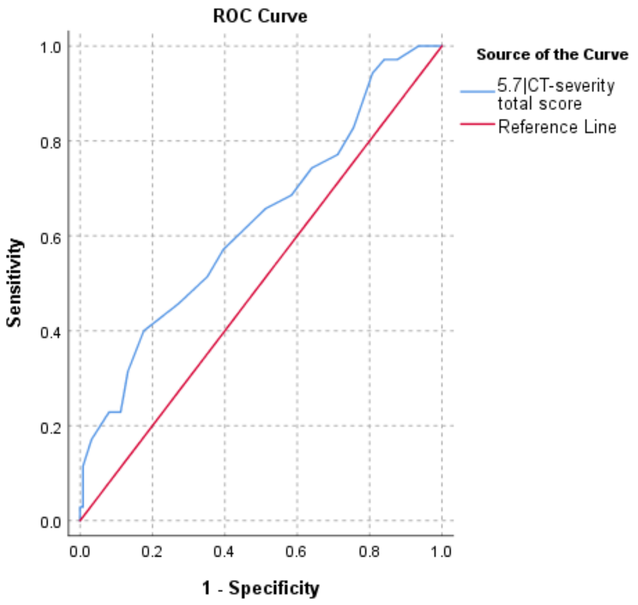

AUC: 0.63 (CI 0.527-0.733); Ideal cut-off point: 13 (sens: 72.2; spec: 79.6)

e-Figure 4, Grey zone of CTSS vs hospital admission, ICU admission and 30-day mortality

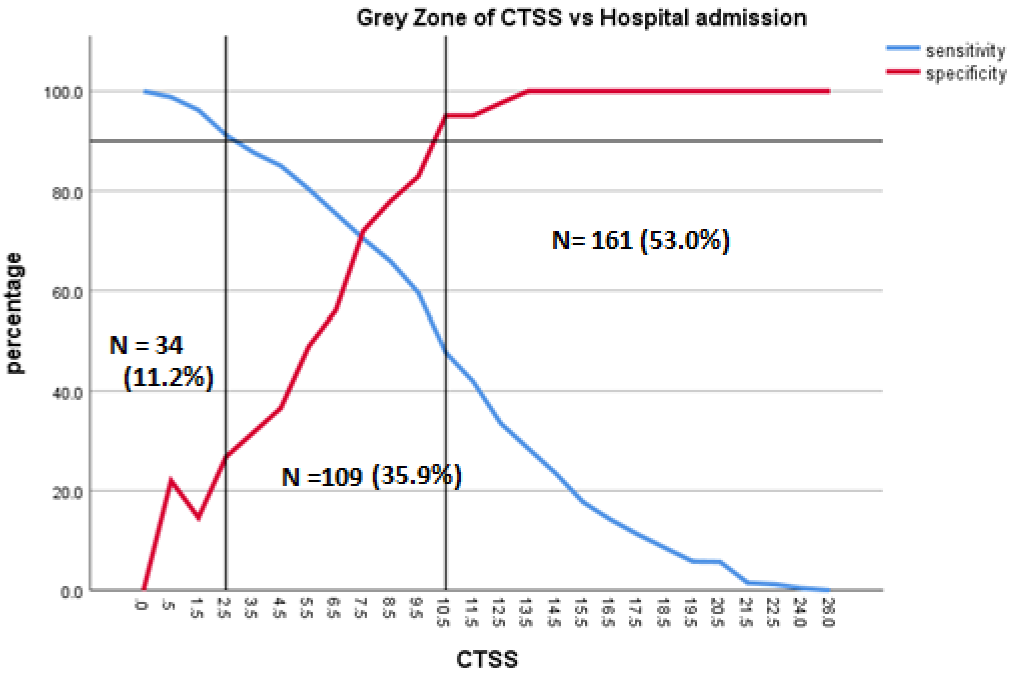

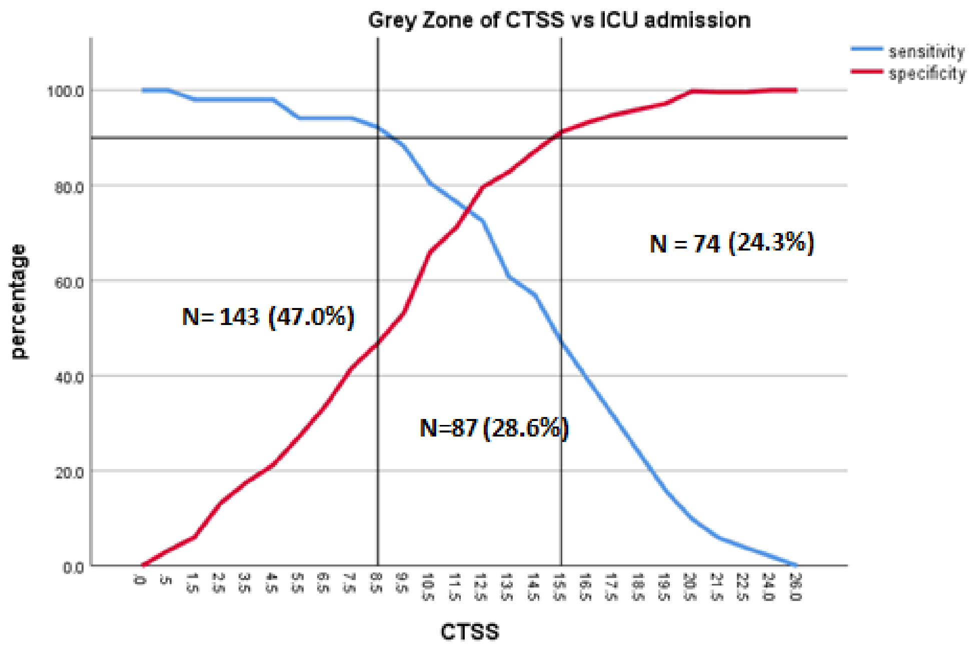

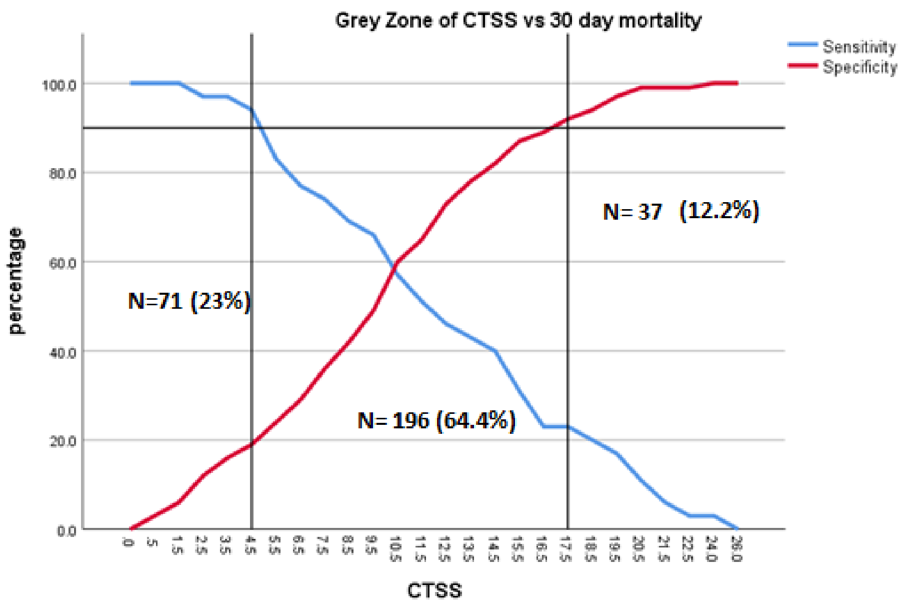

Online supplements are not copyedited prior to posting and the author(s) take full responsibility for the accuracy of all data.

e-Table 1: CO-RADS scoring system, after Prokop et al, 2020.

Score	Level of suspicion	Pulmonary findings	Obligatory features	Confirmatory patterns	Examples (of alternative diagnoses)
0	Not interpretable	• Incomplete depiction of the lung on CT OR<br>• Technically insufficient for assigning a classification			Severe breathing or coughing artifacts
1	Very low	Normal chest CT	• Findings of unequivocal non-infectious etiology<br>• Findings are stable compared to pathology on previous imaging		• Emphysema<br>• (Known) interstitial pneumonitis<br>• Nodules<br>• Tumor<br>• Interstitial edema
2	Low	• Findings typical of infectious etiology AND considered not compatible with COVID-19 AND<br>• Absence of features of LEVEL 3-5	• Tree-in-bud sign<br>• Centrilobular nodular pattern<br>• Lobar or segmental consolidation<br>• Cavitation		• Bronchitis<br>• Infectious bronchiolitis<br>• Bronchopneumonia<br>• Lobar pneumonia<br>• Pulmonary abscess
3	Equivocal / unsure	• Equivocal for COVID-19<br>• Overlap with other pathology.<br>• Findings have to be new or increased in magnitude	• Perihilar ground-glass<br>• Ground-glass together with smooth interlobular septal thickening +/- pleural effusion<br>• Extensive homogeneous ground-glass opacity<br>• Small ground glass opacities, not centrilobular, not located close to the visceral pleura<br>• Consolidation compatible with organizing pneumonia without other typical findings of COVID-19		• Influenza<br>• RSV or other viral pneumonias<br>• Atypical alveolar edema<br>• Pulmonary hemorrhage<br>• Alternative infections combined with SARS-CoV-2

Online supplements are not copyedited prior to posting and the author(s) take full responsibility for the accuracy of all data.

4	High	• Findings typical for pulmonary involvement of COVID-19 with additional non-typical features seen with other (viral) pneumonia<br>• Findings have to be new or increased Findings similar to LEVEL 5 *but:*	• Predominantly no contact with visceral pleura OR<br>• Located strictly unilaterally OR<br>• Predominant peri-bronchovascular distribution OR<br>• Superimposed on severe diffuse pre-existing pulmonary abnormalities		
5	Very High	• Findings typical for pulmonary involvement of COVID-19<br>• Findings have to be new OR increased	• Ground-glass with or without consolidations close to visceral pleural surfaces, including fissures AND<br>• Bilateral OR multifocal	• Ground glass:<br>-**Unsharp demarcation**, (half) rounded shape.<br>-**Sharp demarcation**, outlining multiple adjacent secondary pulmonary lobules.<br>• Crazy paving.<br>• Patterns compatible with **organizing pneumonia**, such as:<br>-Reverse halo sign,<br>-Extensive subpleural consolidations with air bronchograms<br>-subpleural curvilinear bands<br>-Ground glass with or without consolidation in an arching, tethered pattern with small connections to the pleura.<br>• **Thickened vessels** within abnormalities	
6	PCR positive	Any pulmonary findings			

Online supplements are not copyedited prior to posting and the author(s) take full responsibility for the accuracy of all data.

e-Table 2: CT severity score: min 0 points – max 25 points.

CT severity score per lobe	Points
0%	0
1-5%	1
5-25%	2
25-50%	3
50-75%	4
>75%	5

e-Table 3 Patient Characteristics Excluded patients	Excluded patients N = 419	SARS-CoV-2 PCR negative N = 326	SARS-CoV-2 PCR positive N = 83	P value
Age, mean (SD)	55.6 (17.8)	55.1 (17.9)	57.1 (16.3)	0.101
Male, n (%)	219 (52.3)	170 (52.1)	46 (55.4)	0.194
Admission, n (%)	150 (35.8)	103 (31.6)	45 (54.2)	**0.001**
Admission IC, n (%)	22 (5.3)	14 (4.3)	8 (9.6)	**0.000**
30 days mortality, n (%)	30 (7.2)	21 (6.4)	9 (10.8)	**0.039**
In hospital Mortality, n (%)	23 (5.5)	15 (4.6)	8 (9.6)	**0.000**
Duration of symptoms, days (SD)	7 (9.4)	6.3 (7.3)	8.2 (7.4)	0.181
**Co-morbidities, n (%)**				
Asthma	48 (11.5)	38 (11.7)	8 (9.6)	0.293
Chronic Cardiovascular Disease	71 (16.9)	57 (17.5)	12 (14.5)	0.178
COPD (GOLD >2)	57 (13.6)	47 (14.4)	10 (12.0)	0.258
Current Malignancy	64 (15.3)	61 (18.7)	3 (3.6)	**0.000**
Diabetes Mellitus	55 (13.1)	45 (13.8)	8 (9.6)	**0.037**
Hypertension	105 (25.1)	80 (24.5)	21 (25.3)	0.777
**Observations at presentation**				
Modified early warning score mean (SD)	2.1 (1.6)	2.1 (1.6)	2.2 (1.6)	0.957
Temperature (Celsius), mean (SD)	37.7 (1.0)	36.7 (1.0)	37.1(1.0)	**0.000**
Respiratory rate, mean (SD)	19.2 (5.9)	18.8 (5.6)	20.7 (6.6)	0.091
Saturation levels, mean (SD)	96.2 (4.6)	96.2 (4.7)	95.7 (4.2)	0.912
Oxygen therapy, n (%)	47 (11.2)	28 (8.8)	19 (23.8)	**0.000**
Intubation, n (%)	24 (5.7)	15 (4.6)	9 (10.8)	**0.000**

Online supplements are not copyedited prior to posting and the author(s) take full responsibility for the accuracy of all data.

e-Table 4: Duration of symptoms

CT	**PCR +**<br>**Days, mean (SD)**	**PCR -**<br>**Days, mean (SD)**
CORADS 1	2.7 (1.9)	5.8 (8.9)
CORADS 2	1.8 (1.0)	5.5 (6.7)
CORADS 3	6.1 (6.5)	5.7 (9.0)
CORADS 4	7.1 (4.6)	6.4 (5.6)
CORADS 5	8.5 (4.4)	9.7 (5.7)

e-Table 5: False positive diagnoses

**Diagnoses**	**N (%)**
Bacterial pneumonia	7 (10.8)
Urosepsis	2 (3.1)
Exacerbation COPD	2 (3.1)
Malaise	2 (3.1)
Organ failure in acute decompensated liver cirrhosis	1 (1.5)
Taxol pneumonitis	1 (1.5)
Lymphoma	1 (1.5)
Cellulitis	1 (1.5)
Decompensated heart failure	1 (1.5)
Pericarditis	1 (1.5)

e-Table 6: Alternative diagnoses

**Alternative diagnoses**	**Percentage (%)**
Bacterial/aspiration pneumonia	25
Decompensated heart failure	12
Exacerbation COPD/asthma	10
Malignancy progression	8.5
Upper respiratory tract infection	7.6
Fever of unknown origin	4.0
Dyspnea of unknown origin	3.1
Pulmonary embolism	2.8
Malaise	2.7
Pericarditis	2.2
Sickle cell crisis	2.0
Neurological diseases**	2.0
Chronic cough	1.8
Gastrointestinal bleeding	1.3
Abdominal & gastrointestinal infections*	15

*Group consisting of a broad array of diagnoses: UTI, splenic abscess, abscess after low anterior resection, gastritis, liver cirrhosis, cholangitis, cholecystitis

**Group consisting of a broad array of diagnoses: epilepsy, CVA, intracranial bleeding

Online supplements are not copyedited prior to posting and the author(s) take full responsibility for the accuracy of all data.

e-Figure 5: CUSUM charts

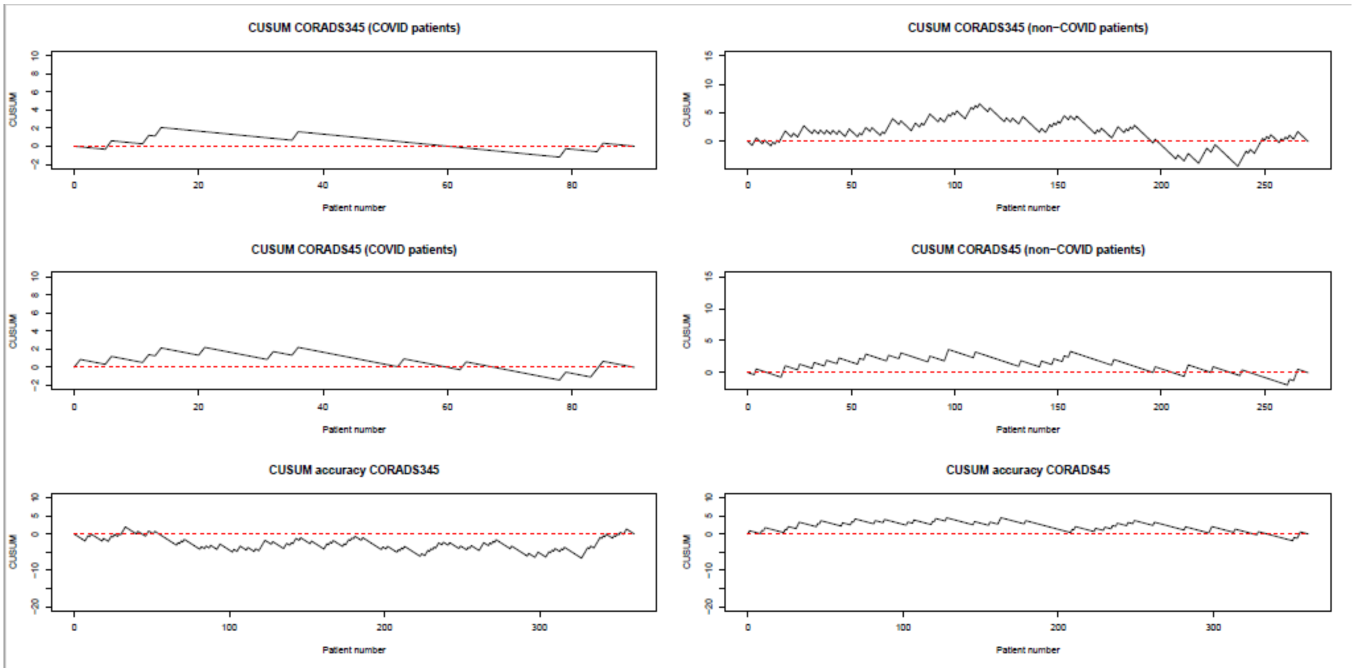

Chart Center 1

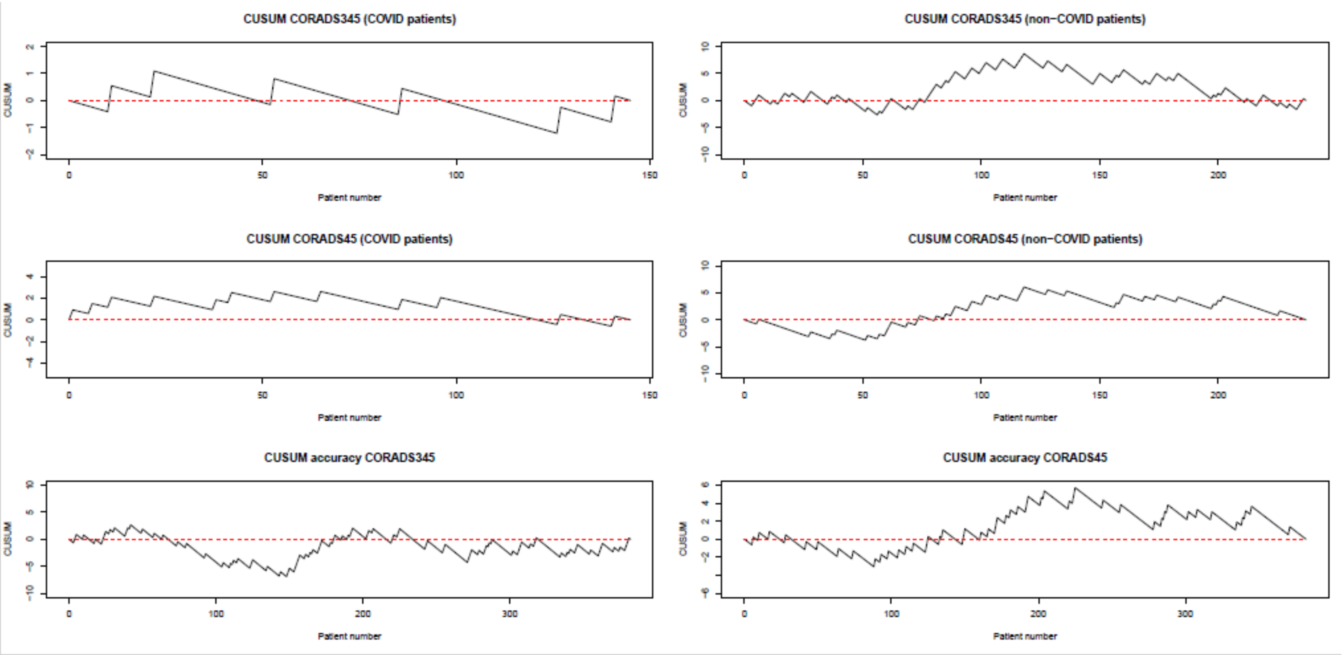

Chart Center 2

Online supplements are not copyedited prior to posting and the author(s) take full responsibility for the accuracy of all data.

e-Appendix 1.

e-Methods

CT-scan

CT scans were performed without the use of intravenous contrast agents in supine position at the end-inspiration level. Four different CT systems were used: GE Discovery CT750 HD (GE Healthcare, Waukesha, WI, USA), Siemens SOMATOM Drive, Siemens SOMATOM Force and Siemens SOMATOM Definition AS+ (Siemens Healthcare, Erlangen, Germany). If necessary, pitch was increased to reduce motion artifacts. Multiplanar CT images are reconstructed with a slice thickness of 0.625 – 3mm.

Online supplements are not copyedited prior to posting and the author(s) take full responsibility for the accuracy of all data.
